# Correction: Critical timing: Impact of delays to surgery on prognosis in stage I-II non-small cell lung cancer

**DOI:** 10.1371/journal.pone.0328912

**Published:** 2025-07-22

**Authors:** The PLOS One Staff

There are errors in the author affiliations. The correct affiliations are as follows:

Ye Zhang^1^, Yeji Hu^1^, Jinfeng Xi^1^, Bo Wu^2^, Wenxiong Zhang^2^, Chunling Li^3^

1 Department of Thoracic Surgery, Jiangxi Provincial People’s Hospital, The First Affiliated Hospital of Nanchang Medical College, Nanchang, China, 2 Department of Thoracic Surgery, The Second Affiliated Hospital, Jiangxi Medical College, Nanchang University, Nanchang, China, 3 Operating Room, Jiangxi Provincial People’s Hospital, The First Affiliated Hospital of Nanchang Medical College, Nanchang, China.

The publisher apologizes for the error.
