# Supplementary figures and images for: Critical timing: Impact of delays to surgery on prognosis in stage I-II non-small cell lung cancer
Source: PLoS One. 2025 May 28;20(5):e0319357. doi: 10.1371/journal.pone.0319357 (PMC12118990; doi:10.1371/journal.pone.0319357)

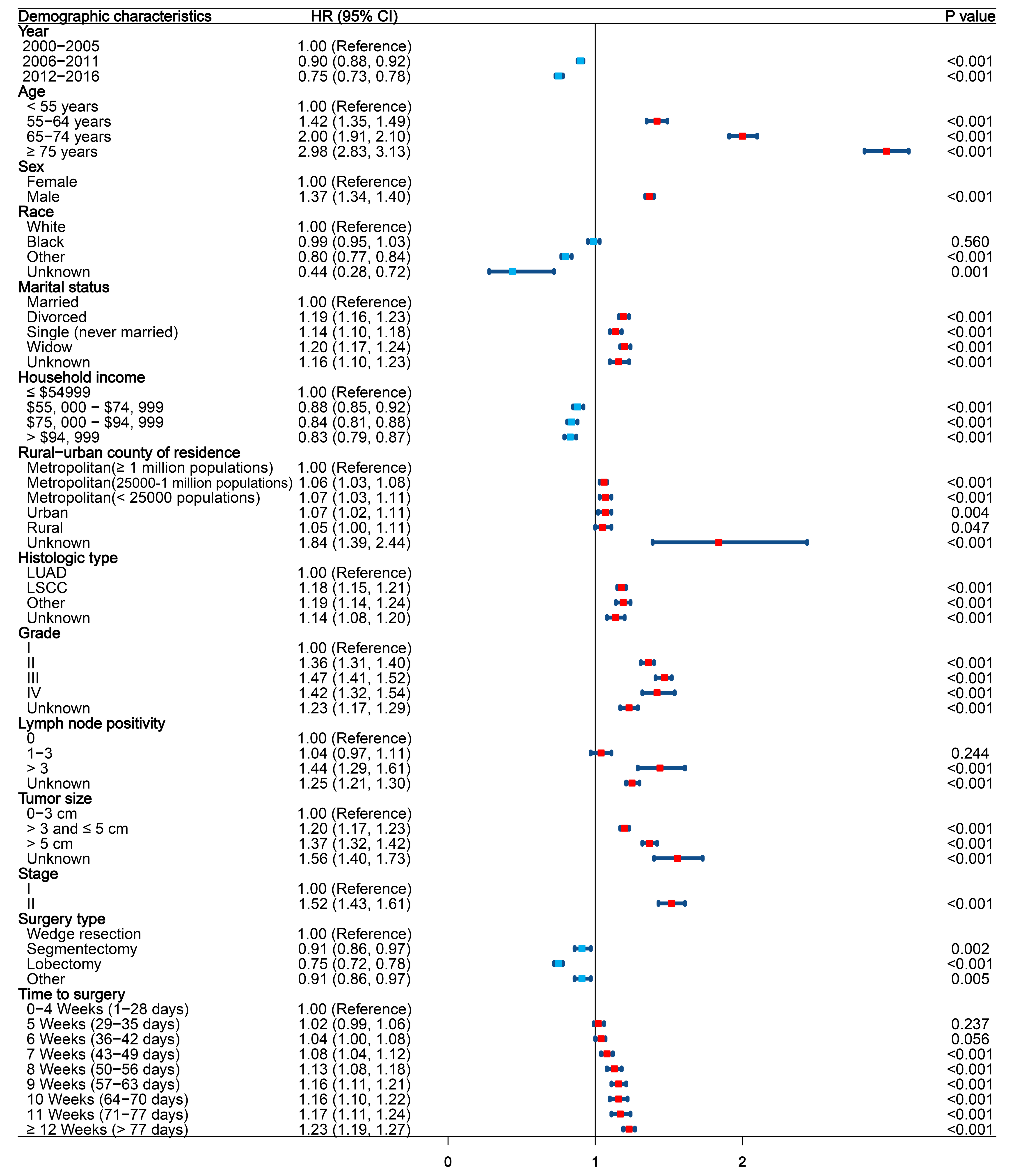

Supplement: Fig S1 — (TIF) [file pone.0319357.s001.tif]

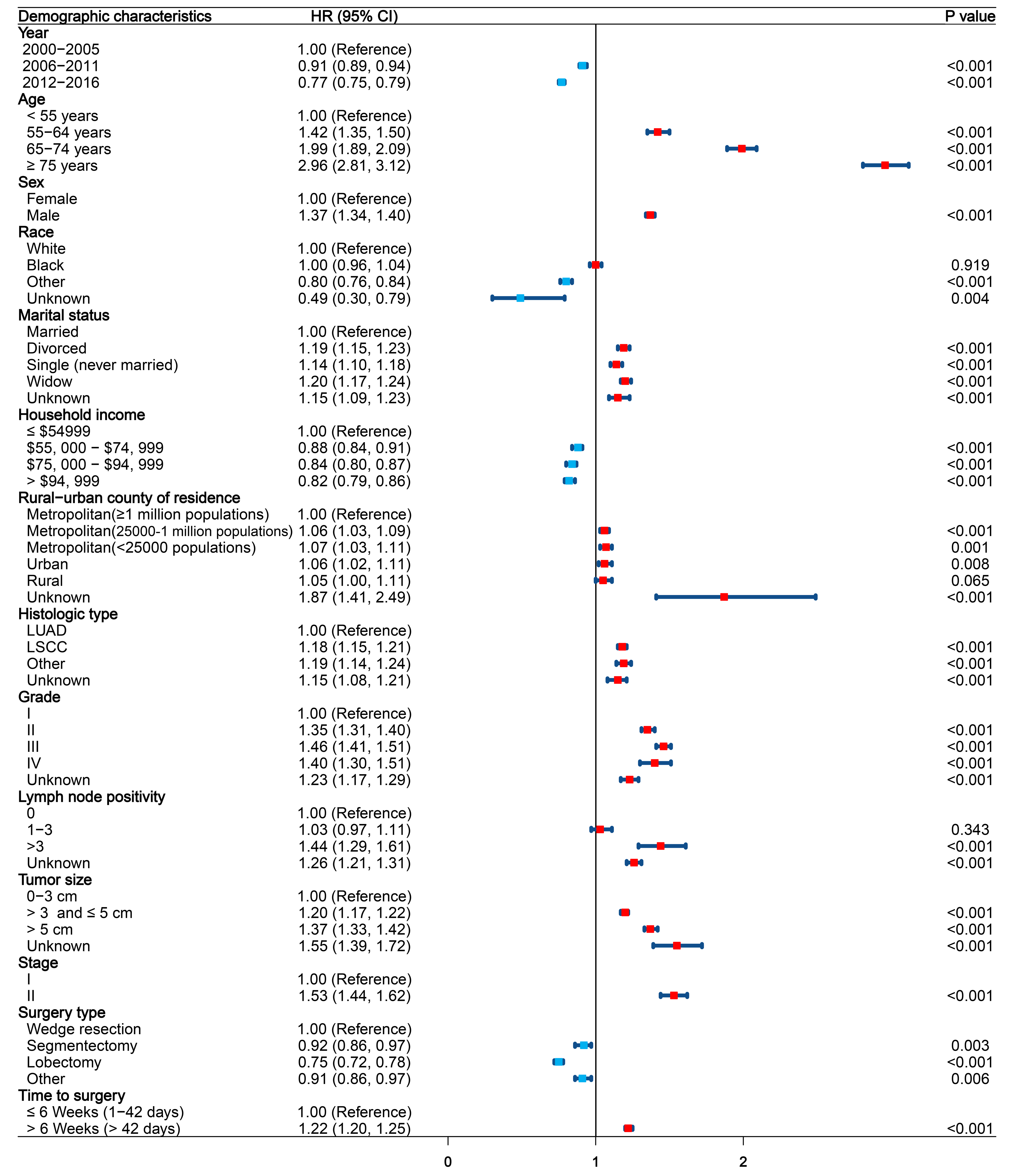

Supplement: Fig S2 — (TIF) [file pone.0319357.s002.tif]

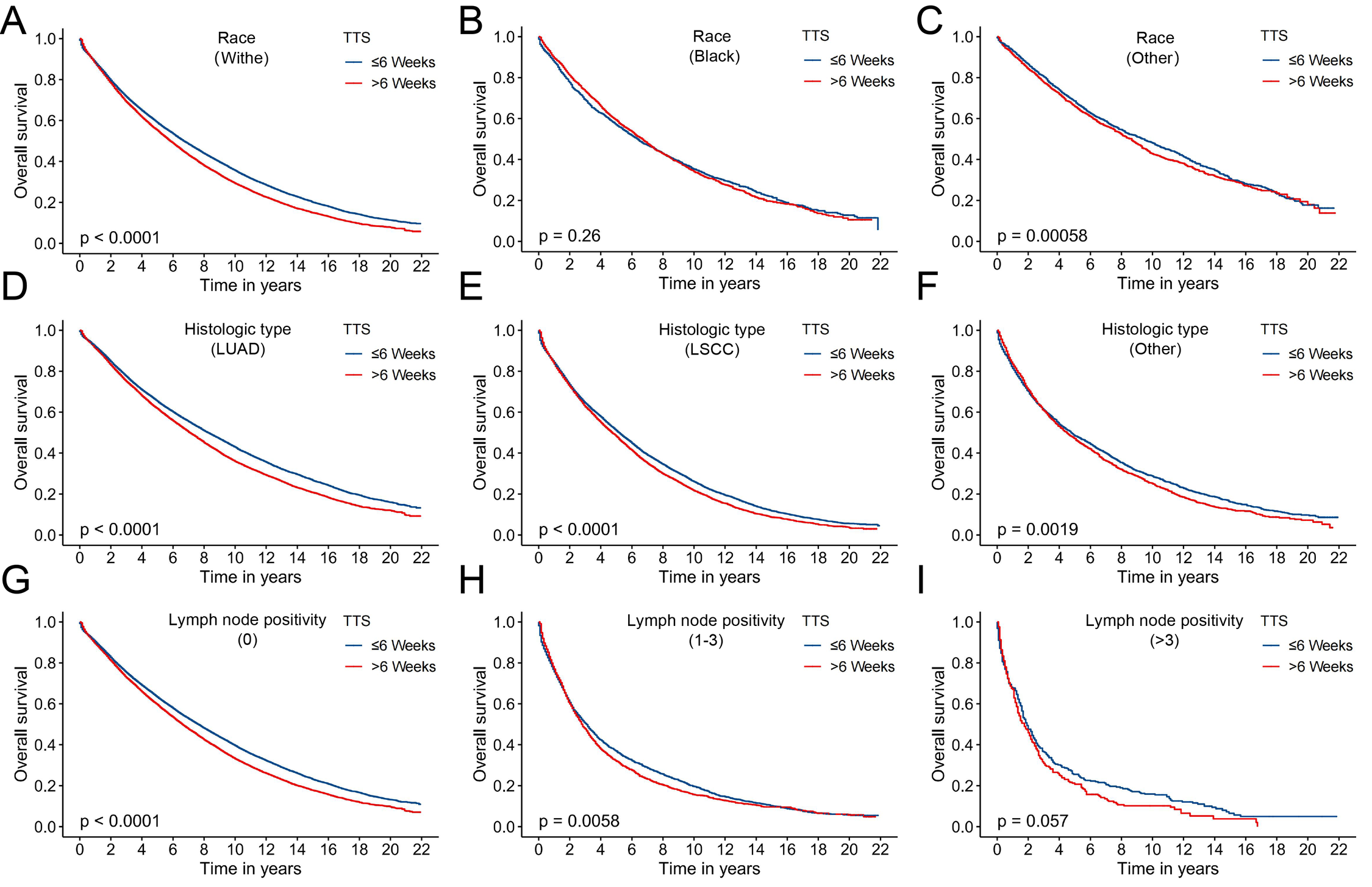

Supplement: Fig S3 — (A) race (White), (B) race (Black), (C) race (other), (D) histologic type (LUAD), (E) histologic type (LSCC), (F) histologic type (other), (G) lymph node positivity (0), (H) lymph node positivity (1–3), (I) lymph node positivity (> 3). (TIF) [file pone.0319357.s003.tif]

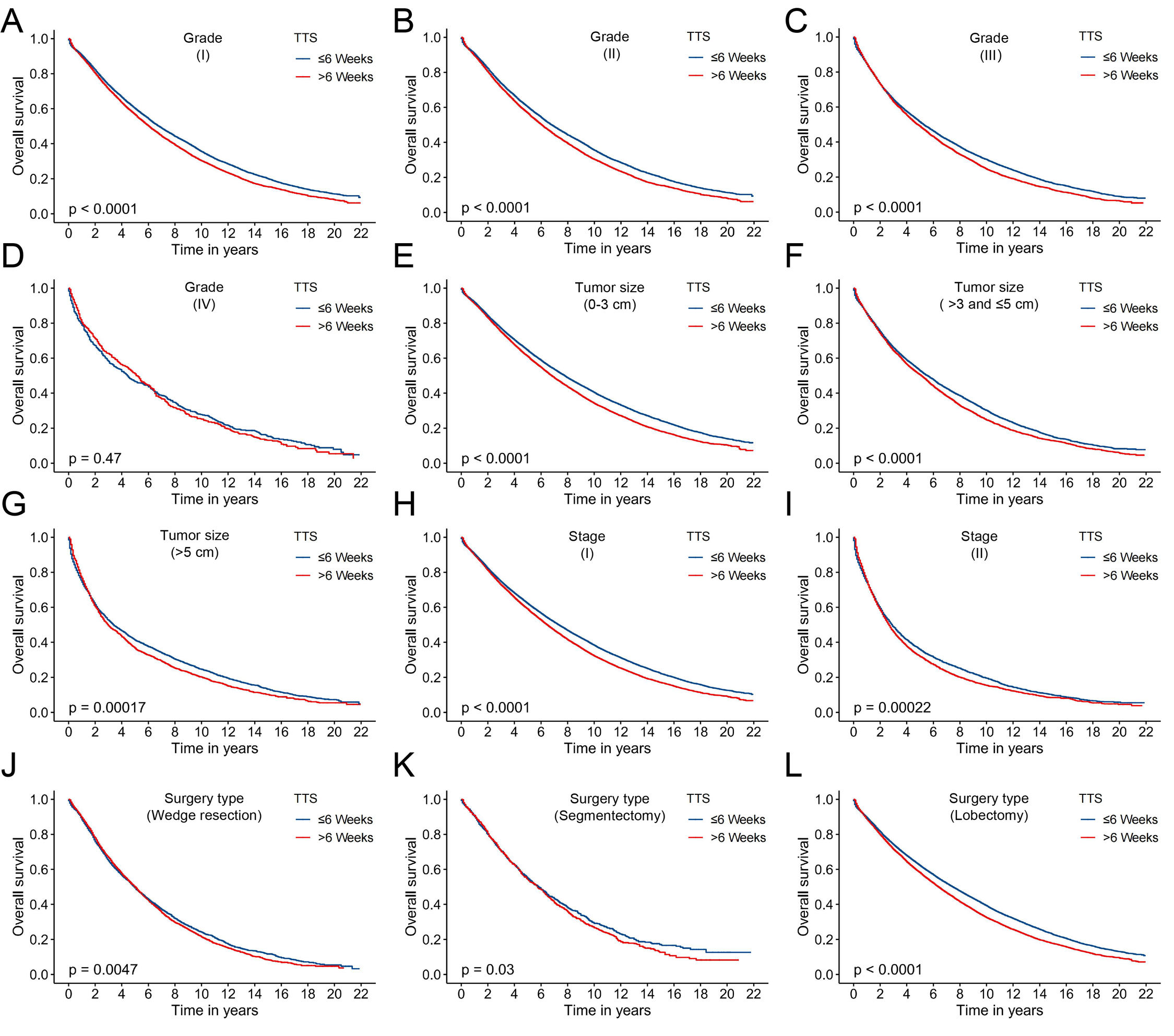

Supplement: Fig S4 — (A) grade (I), (B) grade (II), (C) grade (III), (D) grade (IV), (E) tumor size (0–3 cm), (F) tumor size (> 3 cm and ≤ 5 cm) and (G) tumor size (> 5 cm), (H) stage (I), (I) stage (II), (J) surgery type (wedge resection), (K) surgery type (segmentectomy) and (L) surgery type (lobectomy). (TIF) [file pone.0319357.s004.tif]
