# Supplementary material for: Critical timing: Impact of delays to surgery on prognosis in stage I-II non-small cell lung cancer
Source: PLoS One. 2025 May 28;20(5):e0319357. doi: 10.1371/journal.pone.0319357 (PMC12118990; doi:10.1371/journal.pone.0319357)
Supplement: Table S1 — (DOCX) [file pone.0319357.s005.docx]

Table S1 Characteristics of stage I-II NSCLC patients before and after IPTW in the TTS ≤ 6 weeks and TTS > 6 weeks groups.

| **Demographic characteristics** | **Unadjusted** | | |  | **IPTW-adjusted** | | |
| --- | --- | --- | --- | --- | --- | --- | --- |
|  | **≤ 6 Weeks**  **(N=29985)** | **> 6 Weeks (N=25597)** | **P value** |  | **≤ 6 Weeks (Weights=27801)** | **> 6 Weeks (Weights=27781)** | **P value** |
| **Year of diagnosis** |  |  | < 0.001 |  |  |  | 0.999 |
| 2000-2005 | 9660 (32.22%) | 6849 (26.76%) |  |  | 29.70% | 29.70% |  |
| 2006-2011 | 9570 (31.92%) | 8243 (32.2%) |  |  | 32.00% | 32.00% |  |
| 2012-2016 | 10755 (35.87%) | 10505 (41.04%) |  |  | 38.30% | 38.30% |  |
| **Age** |  |  | < 0.001 |  |  |  | 1.000 |
| ≤ 45 years | 2411 (8.04%) | 1576 (6.16%) |  |  | 7.15% | 7.15% |  |
| 55-64 years | 6592 (21.98%) | 5267 (20.58%) |  |  | 21.32% | 21.32% |  |
| 65-74 years | 11884 (39.63%) | 10017 (39.13%) |  |  | 39.42% | 39.42% |  |
| ≥ 75 years | 9098 (30.34%) | 8737 (34.13%) |  |  | 32.22% | 32.22% |  |
| **Sex** |  |  | 0.006 |  |  |  | 0.947 |
| Female | 15380 (51.29%) | 12831 (50.13%) |  |  |  |  |  |
| Male | 14605 (48.71%) | 12766 (49.87%) |  |  | 49.24% | 49.24% |  |
| **Race** |  |  | < 0.001 |  |  |  | 1.000 |
| White | 26221 (87.45%) | 21346 (83.39%) |  |  | 85.54% | 85.54% |  |
| Black | 1888 (6.3%) | 2344 (9.16%) |  |  | 7.64% | 7.64% |  |
| Other | 1838 (6.13%) | 1880 (7.34%) |  |  | 6.71% | 6.71% |  |
| Unknown | 38 (0.13%) | 27 (0.11%) |  |  | 0.12% | 0.12% |  |
| **Marital status** |  |  | < 0.001 |  |  |  | 1.000 |
| Married | 18044 (60.18%) | 13416 (52.41%) |  |  | 56.62% | 56.62% |  |
| Divorced | 3454 (11.52%) | 3573 (13.96%) |  |  | 12.63% | 12.63% |  |
| Single (never married) | 2709 (9.03%) | 2949 (11.52%) |  |  | 10.21% | 10.21% |  |
| Widow | 4751 (15.84%) | 4891 (19.11%) |  |  | 17.43% | 17.43% |  |
| Unknown | 1027 (3.43%) | 768 (3%) |  |  | 3.21% | 3.21% |  |
| **Household income** |  |  | < 0.001 |  |  |  | 0.999 |
| ≤ $54999 | 4110 (13.71%) | 3061 (11.96%) |  |  | 12.83% | 12.83% |  |
| $55,000 - $74,999 | 11259 (37.55%) | 9805 (38.31%) |  |  | 37.87% | 37.87% |  |
| $75,000 - $94,999 | 8243 (27.49%) | 7419 (28.98%) |  |  | 28.20% | 28.20% |  |
| > $94,999 | 6373 (21.25%) | 5312 (20.75%) |  |  | 21.07% | 21.07% |  |
| **Rural-urban county of residence** |  |  | < 0.001 |  |  |  | 1.000 |
| Metropolitan (≥ 1 million populations) | 16455 (54.88%) | 14977 (58.51%) |  |  | 56.67% | 56.67% |  |
| Metropolitan (25000-1 million populations) | 6259 (20.87%) | 5244 (20.49%) |  |  | 20.74% | 20.74% |  |
| Metropolitan (< 25000 populations) | 2758 (9.2%) | 2010 (7.85%) |  |  | 8.54% | 8.54% |  |
| Urban | 2646 (8.82%) | 1972 (7.7%) |  |  | 8.30% | 8.30% |  |
| Rural | 1837 (6.13%) | 1359 (5.31%) |  |  | 5.75% | 5.75% |  |
| Unknown | 30 (0.1%) | 35 (0.14%) |  |  | 0.12% | 0.12% |  |
| **Histologic type** |  |  | < 0.001 |  |  |  | 1.000 |
| LUAD | 18366 (61.25%) | 15192 (59.35%) |  |  | 60.44% | 60.44% |  |
| LSCC | 8667 (28.9%) | 7886 (30.81%) |  |  | 29.78% | 29.78% |  |
| Other | 2107 (7.03%) | 1652 (6.45%) |  |  | 6.80% | 6.80% |  |
| Unknown | 845 (2.82%) | 867 (3.39%) |  |  | 3.10% | 3.10% |  |
| **Grade** |  |  | 0.003 |  |  |  | 1.000 |
| I | 4665 (15.56%) | 3747 (14.64%) |  |  | 15.10% | 15.10% |  |
| II | 12720 (42.42%) | 11059 (43.2%) |  |  | 42.80% | 42.80% |  |
| III | 10044 (33.5%) | 8564 (33.46%) |  |  | 33.40% | 33.40% |  |
| IV | 646 (2.15%) | 496 (1.94%) |  |  | 2.11% | 2.11% |  |
| Unknown | 1910 (6.37%) | 1731 (6.76%) |  |  | 6.56% | 6.56% |  |
| **Lymph node positivity** |  |  | < 0.001 |  |  |  | 1.000 |
| 0 | 25107 (83.73%) | 20989 (82%) |  |  | 82.90% | 82.90% |  |
| 1-3 | 1976 (6.59%) | 1647 (6.43%) |  |  | 6.50% | 6.50% |  |
| > 3 | 276 (0.92%) | 214 (0.84%) |  |  | 0.88% | 0.88% |  |
| Unknown | 2626 (8.76%) | 2747 (10.73%) |  |  | 9.75% | 9.75% |  |
| **Tumor size** |  |  | 0.003 |  |  |  | 1.000 |
| 0-3 cm | 20188 (67.33%) | 16882 (65.95%) |  |  | 66.72% | 66.72% |  |
| > 3 and ≤ 5 cm | 6991 (23.31%) | 6257 (24.44%) |  |  | 23.80% | 23.80% |  |
| > 5 cm | 2558 (8.53%) | 2264 (8.84%) |  |  | 8.64% | 8.64% |  |
| Unknown | 248 (0.83%) | 194 (0.76%) |  |  | 0.80% | 0.80% |  |
| **Stage** |  |  | 0.276 |  |  |  | 0.962 |
| I | 26973 (89.95%) | 22954 (89.67%) |  |  |  |  |  |
| II | 3012 (10.05%) | 2643 (10.33%) |  |  | 10.22% | 10.12% |  |
| **Surgery type** |  |  | < 0.001 |  |  |  | 1.000 |
| Wedge resection | 3433 (11.45%) | 3566 (13.93%) |  |  | 12.72% | 12.72% |  |
| Segmentectomy | 1125 (3.75%) | 1024 (4%) |  |  | 3.89% | 3.89% |  |
| Lobectomy | 24299 (81.04%) | 20263 (79.16%) |  |  | 80.13% | 80.13% |  |
| Other | 1128 (3.76%) | 744 (2.91%) |  |  | 3.38% | 3.38% |  |

**Abbreviations:** IPTW: Inverse probability of treatment weighting; LSCC: Lung squamous cell carcinoma; LUAD: Lung adenocarcinoma; NSCLC: Non-small cell lung cancer.
