# Supplementary material for: Critical timing: Impact of delays to surgery on prognosis in stage I-II non-small cell lung cancer
Source: PLoS One. 2025 May 28;20(5):e0319357. doi: 10.1371/journal.pone.0319357 (PMC12118990; doi:10.1371/journal.pone.0319357)
Supplement: Table S2 — (DOCX) [file pone.0319357.s006.docx]

Table S2 Multinomial logistic regression of 51-75th Percentile (TTS 41-62 days), 75-95th Percentile (TTS 63-111 days) and > 95th Percentile (TTS > 111 days) groups compared to a reference group of patients in the 0 to 50th percentile (TTS 1-40 days).

| **Demographic characteristics** | **OR (95% CI) P value** | | | | | | | |
| --- | --- | --- | --- | --- | --- | --- | --- | --- |
|  | **51-75th Percentile (40-62 days)** | |  | **76-95th Percentile (63-111 days)** | |  | **>95th Percentile (> 111 days)** | |
| **Household income** |  |  |  |  |  |  |  |  |
| ≤ $54999 | 1.00 (Reference) |  |  | 1.00 (Reference) |  |  | 1.00 (Reference) |  |
| $55,000 - $74,999 | 1.07 (0.99, 1.16) | 0.105 |  | 1.13 (1.03, 1.24) | 0.008 |  | 1.08 (0.94, 1.24) | 0.298 |
| $75,000 - $94,999 | 1.13 (1.03, 1.24) | 0.009 |  | 1.16 (1.05, 1.29) | 0.004 |  | 1.10 (0.94, 1.29) | 0.221 |
| > $94,999 | 1.03 (0.94, 1.14) | 0.496 |  | 1.09 (0.98, 1.21) | 0.125 |  | 1.01 (0.85, 1.19) | 0.920 |
| **Rural-urban county of residence** |  |  |  |  |  |  |  |  |
| Metropolitan (≥ 1 million populations) | 1.00 (Reference) |  |  | 1.00 (Reference) |  |  | 1.00 (Reference) |  |
| Metropolitan (25000-1 million populations) | 0.92 (0.87, 0.97) | 0.003 |  | 0.90 (0.85, 0.95) | < 0.001 |  | 0.92 (0.84, 1.01) | 0.065 |
| Metropolitan (< 25000 populations) | 0.87 (0.80, 0.94) | 0.001 |  | 0.74 (0.67, 0.81) | < 0.001 |  | 0.84 (0.73, 0.97) | 0.019 |
| Urban | 0.86 (0.78, 0.94) | 0.001 |  | 0.88 (0.79, 0.97) | 0.013 |  | 0.86 (0.74, 1.02) | 0.078 |
| Rural | 0.88 (0.79, 0.99) | 0.027 |  | 0.87 (0.77, 0.98) | 0.024 |  | 0.87 (0.72, 1.06) | 0.157 |
| Unknown | 1.08 (0.58, 2.02) | 0.808 |  | 1.32 (0.70, 2.47) | 0.389 |  | 1.10 (0.42, 2.92) | 0.841 |
| **Histologic type** |  |  |  |  |  |  |  |  |
| LUAD | 1.00 (Reference) |  |  | 1.00 (Reference) |  |  | 1.00 (Reference) |  |
| LSCC | 1.06 (1.01, 1.12) | 0.014 |  | 1.10 (1.05, 1.17) | < 0.001 |  | 1.00 (0.91, 1.08) | 0.909 |
| Other | 0.92 (0.84, 1.01) | 0.072 |  | 1.05 (0.95, 1.16) | 0.303 |  | 0.90 (0.77, 1.05) | 0.181 |
| Unknown | 1.31 (1.16, 1.48) | < 0.001 |  | 1.17 (1.02, 1.34) | 0.026 |  | 1.31 (1.07, 1.59) | 0.009 |
| **Grade** |  |  |  |  |  |  |  |  |
| I | 1.00 (Reference) |  |  | 1.00 (Reference) |  |  | 1.00 (Reference) |  |
| II | 1.07 (1.00, 1.14) | 0.040 |  | 1.07 (1.00, 1.15) | 0.064 |  | 1.01 (0.91, 1.13) | 0.799 |
| III | 1.04 (0.97, 1.11) | 0.263 |  | 1.08 (1.00, 1.16) | 0.054 |  | 0.98 (0.88, 1.11) | 0.795 |
| IV | 1.01 (0.86, 1.19) | 0.883 |  | 0.94 (0.78, 1.14) | 0.543 |  | 0.85 (0.63, 1.15) | 0.300 |
| Unknown | 1.05 (0.95, 1.16) | 0.355 |  | 1.14 (1.03, 1.28) | 0.016 |  | 1.44 (1.23, 1.68) | < 0.001 |
| **Lymph node positivity** |  |  |  |  |  |  |  |  |
| 0 | 1.00 (Reference) |  |  | 1.00 (Reference) |  |  | 1.00 (Reference) |  |
| 1-3 | 0.98 (0.85, 1.14) | 0.823 |  | 0.90 (0.77, 1.06) | 0.228 |  | 0.98 (0.76, 1.26) | 0.866 |
| > 3 | 0.97 (0.75, 1.24) | 0.798 |  | 0.86 (0.65, 1.13) | 0.279 |  | 0.89 (0.57, 1.38) | 0.600 |
| Unknown | 1.08 (1.00, 1.16) | 0.044 |  | 1.30 (1.21, 1.41) | < 0.001 |  | 1.61 (1.45, 1.80) | < 0.001 |
| **Tumor size** |  |  |  |  |  |  |  |  |
| 0-3 cm | 1.00 (Reference) |  |  | 1.00 (Reference) |  |  | 1.00 (Reference) |  |
| > 3 and ≤ 5 cm | 1.09 (1.04, 1.15) | 0.001 |  | 1.08 (1.02, 1.14) | 0.009 |  | 1.01 (0.92, 1.10) | 0.831 |
| > 5 cm | 1.08 (1.00, 1.17) | 0.058 |  | 1.08 (0.99, 1.18) | 0.084 |  | 1.02 (0.89, 1.17) | 0.733 |
| Unknown | 0.95 (0.75, 1.20) | 0.678 |  | 0.88 (0.67, 1.14) | 0.339 |  | 0.83 (0.55, 1.24) | 0.358 |
| **Stage** |  |  |  |  |  |  |  |  |
| I | 1.00 (Reference) |  |  | 1.00 (Reference) |  |  | 1.00 (Reference) |  |
| II | 1.05 (0.92, 1.20) | 0.451 |  | 1.10 (0.95, 1.26) | 0.195 |  | 1.10 (0.89, 1.37) | 0.365 |

**Abbreviations:** CI: Confidence interval; LSCC: Lung squamous cell carcinoma; LUAD: Lung adenocarcinoma; NSCLC: Non-small cell lung cancer; OR: Odds ratio.
